# A short review on behavioural assessment methods in rodents

**DOI:** 10.6026/97320630019866

**Published:** 2023-08-31

**Authors:** Angu Bala Ganesh K.S.V., Dilshani Dissanayake, Mangala Gunatilake, Venkatapathy Kuzhandai Velu, Modagan Paranthaman

**Affiliations:** Department of Anatomy, CU Shah Medical College and Hospital, Dudhrej Road, Surendranagar 363001, Gujarat, India; Department of Physiology, Faculty of Medicine, University of Colombo, No. 25, Kynsey road, Colombo 08, Sri Lanka; Department of Biochemistry, Mahatma Gandhi Medical College and Research Institute, SBV, Puducherry 607 402, India; Department of Biochemistry, Dhanalakshmi srinivasan Medical college, Perambalur, Tamil Nadu 621212, India

**Keywords:** Rodents, behavioural, cognitive, motor, social activities

## Abstract

The rodent behavioural examination techniques are used to assess various psychological, neurological models and neurotoxicity studies. Therefore, it is of interest to document the various behavioural assessment methods used in rodent model to study the motor, sensory, cognitive functions and emotional behaviour.

## Background:

Over the past decade, there has been a steady increase in the study of laboratory animal behavior. This field has placed a growing emphasis on various commonly used species, recognizing the significance of animals' motor, cognitive, and social activities. [1] Despite being kept in captivity for extended periods, laboratory animals retain their instinctual behaviors and characteristics that evolved in their wild ancestors. It is essential to test their natural active behaviors, such as exploration, inquisitiveness, and digging, aggression, rearing, and climbing, while considering well-defined circadian rhythms. [2] Behavior assessment is a scientific approach that relies on the ability of human observers to integrate observed details of behavior, posture, and context to summarize animals' behavioral patterns. [3] Observers use descriptors like "relaxed," "anxious/tense," "frustrated," or "content" to evaluate and assess animals' emotional states. The use of feasible indicators to evaluate animals' emotional states is strongly recommended in behavioral assessments. [4] Behaviors can be measured in terms of interval (also known as latency), frequency, and duration. Interval measures the time it takes for a specific behavior to occur. It can be measured in seconds, minutes, or hours. Frequency refers to the number of times a behavior occurs during an observation period and is usually measured per minute, per hour, or per day. Duration measures how long a behavior lasts and can be measured in seconds, minutes, or hours. [5] Rodent models are essential for studying brain disorders, including neurodevelopmental, neuropsychiatric, and neurodegenerative diseases. They help increase our understanding of underlying pathology and serve as preclinical models for testing potential treatments. [6] Behavioral outcomes are among the most significant measures in these studies. Unfortunately, reports from different laboratories often yield conflicting results, and findings from rodent models are not consistently replicated in human trials [7]. There are several well-established tests available to assess various behavioural readouts. However, subtle aspects such as housing conditions, testing conditions, and the sex and strain of animals can influence the measurements. [8] Therefore, it is important to consider these factors during behavioral testing to ensure the reliability and reproducibility of results. Zhang *et al.* in 2017 used a chronic migraine rat model to evaluate depression and anxiety behavior using a panel of tests. [9] The group that received inflammatory soup infusion showed a decrease in sucrose preference, locomotor and rearing behaviors, inner zone distance percent, open-arm entry percent, and serotonin and dopamine levels in the prefrontal brain. [10] Weight, inner zone time percentage, or open-arm time percentage weren't different between the two groups. Therefore, it is of interest to document the various behavioural assessment methods used in rodent model to study the motor, sensory, cognitive functions and emotional behaviour. cognitive and behavioral in rodent models of brain aging, dementia and various behavioral paradigms, such as the Y maze, Morris water maze, Barnes maze, and more, were discussed. These paradigms are used to test spatial memory, recognition memory, semantic memory, spatial memory, and emotional memory [11].

## Tests to assess the motor function and coordination:

Table 1 presents various behavioral tests for evaluating motor function and coordination. Tests include the staircase, beam walking, rotarod, open field, cylinder, foot-fault, pole, and elevated body swing tests for muscle strength. Evaluation of fine motor coordination and balance involved tests like staircase, rotarod, cylinder, grid walk, and beam walking. Tests such as forelimb placing identified forelimb function and deficits, while the wire hanging test evaluated locomotor abnormalities and behavioral deficits [12]. The climbing test assessed motor impairments, and the grid stepping evaluated sensorimotor deficits. Grip strength was measured to assess skeletal muscle function, and skilled limb use evaluated voluntary motor control. Reaching tests and the acoustic startle response assessed motor and cognitive performance. Operant tasks were used for cognitive performance evaluation [13]. Tests like the rotarod, triple horizontal bar, static rods, and parallel bar evaluated muscle coordination and strength [14]. Foot fault assessed motor functions, and the pole test evaluated movement disorders [9]. Neuromuscular weakness was assessed through grip strength, swimming tests measured endurance, and gait analysis identified ataxic and paretic gait. Various methods were applied for assessing motor coordination, balance, and rigidity [15].

## Tests to assess sensory function:

Table 2 outlines methods for testing sensory functions in rodents, including sensory assessment and sensorimotor behavior. Tests for sensory neglect and sensorial limb placement comprised the adhesive removal and limb placement tests. The corner test evaluated sensorimotor and postural asymmetries, while adhesive removal identified tactile responses and asymmetries [9, 16]. Mechanical allodynia measured pain response, and thermal hyperalgesia assessed heat thresholds. Cold hyperalgesia tests were conducted for cold allodynia and hyperalgesia [12]. Von Frey hair and sticky dot tests were employed for sensory and sensorimotor function evaluation [7]. Chemical stimuli were evaluated using the formalin pain test, hot and cold temperature tests and mechanical stimuli were measured through the Von Frey and Randall Selitto tests. [17] Heat stimuli were assessed with tail flick and hot plate tests, and cold stimuli with cold plate and acetone evaporation tests. Paw withdrawal, acoustic startle, and sensorimotor gating tests assessed escape behavior [14].

## Test to assess cognitive (learning and memory) functions:

Table 3 lists methods for testing cognitive (learning and memory) functions of rodents. Tests included the Morris water maze, radial arm maze, and modified elevated plus-maze for spatial learning and memory. Passive avoidance and the three-panel runway maze assessed avoidance learning and working memory [18]. The Y-maze test evaluated active working short-term memory, while the object recognition test assessed various memory types [16]. Barnes maze, eight-arm radial maze, and swimming pool spatial tasks were used for memory acquisition assessment [13]. T-maze and operant chamber tests assessed short-term memory and learned performance [14]. Radial arm water maze and Y-maze spontaneous alternation task evaluated spatial working memory [11]. Novel object recognition and fear conditioning assessed associative and working memory [19]. Olfactory memory and learning were measured through odor preference conditioning [14].

## Test to assess emotional state:

Table 4 provides methods for assessing the emotional state of rodents, including anxiety and depression symptoms. Tests for anxiety-like behavior encompassed open field, novelty suppressed feeding, and raised plus maze tests. The light/dark box test evaluated anxiety-related behavior [20]. Elevated zero maze and marble burying tests were employed for anxiety-related behaviors, and the hole-board test measured anxiety, stress and emotion. Tests to assess emotion and depression included the forced swim test, sucrose preference, and tail suspension test [7]. Coated and splash tests evaluated depression-like behavior. Upraised plus maze and forced swim tests assessed depression [21]. Tests such as the star maze and hole-board maze were used to measure emotional state [15]. Novelty-suppressed feeding and forced swim tests were employed for depression assessment [7].

## Conclusion:

Behavioural assessment is a critical parameter in neurotoxicity studies, drug development, and neurological and psychological model screening. A wide range of tests are available for assessing motor, sensory, cognitive, and emotional states in rodents. The choice of tests should align with the study's nature and be carried out with adherence to ethical animal handling practices. To minimize bias during analysis, video recordings of behavioral sessions are recommended.

## Figures and Tables

**Table 1:** List of various behavioural tests to assess the motor function and coordination

**Name of the Behavioral Test**	**To Identify**
**Beam Walking**	Motor Coordination
**Rotarod**	Coordination of Muscle And Balance
**Open Field**	Locomotion
**Elevated Body Swing**	Strength of the Muscle
**Cylinder Test**	Motor Coordination
**Flexion of Forelimb**	Forelimb Function
**Placing of Forelimb**	Forelimb Function and Slippage
**Grid Walking**	Motor Coordination and Placing Deficits During Locomotion
**Ledged Tapered Beam**	Hind Limb Functioning
**Reaching Chamber/Pellet Retrieval**	Skilled Forepaw Use and Motor Functioning
**Staircase**	Forelimb Extension, Grasping Skills, Side Bias and Independent Use of Forelimbs
**Pasta**	Manual Dexterity and Fine Motor Skills
**Ladder Rung Walking**	Fore- and Hind Limb Stepping, Placing And Coordination
**Wire Hanging**	Abnormalities in Locomotion and behavioral Deficits in Models
**Horizontal Ladder**	Walking Ability
**Swimming**	Coordinated Limb Use During Voluntary Locomotion
**Climbing**	Monitor Motor Impairments
**Grid Stepping**	Assess of Sensoromotor Deficit
**Skilled Limb Use**	Analyze Finer Aspects of Voluntary Motor Control
**Descriptive Paw Use**	Characterization of Fine Motor Control
**Reaching Tests**	Skilled Forepaw Use and Motor Functioning
**Acoustic Startle Response**	Muscular Activity Produced in Response to a Sudden Loud Sound
**Operant Tasks**	Motor and Cognitive Performance in Rats and Mice
**Sequence Learning**	Motor Habit Learning Task
**Triple Horizontal Bar**	Measure Motor Coordination and Strength
**Static Rods**	Measure Motor Coordination
**Parallel Bars**	Measuring Motor Capabilities
**Foot-Fault Test**	Assess Motor Functions
**Pole Test**	Assess Movement Disorders in Mice
**Grip Strength**	Neuromuscular Weakness
**Gait Analysis**	Ataxic and Paretic Gait
**Treadmill, Coordinated Movement Test**	In coordination
**Bar Test**	Catalepsy Bar Test to Measure Rigidity

**Table 2:** List of specific methods in testing sensory function of rodents.

**Name of the Behavioural Test**	**To Identify**
**Limb Placement Test**	Sensorial Limb Placement
**Corner**	Sensorimotor and Postural Asymmetries - Whiskers Sensitivity
**Adhesive Removal Test**	Tactile Responses and Asymmetries
**Whisker Nuisance Task**	Sensorimotor Integration
**Gap Cross Test**	Evaluation of Somatosensory Behaviour
**Angle Entrance Task**	Evaluation of Somatosensory Behaviour
**Whisker Guided Exploration Task**	Somatosensory Behavior
**The Von Frey Hair Test**	Evaluate Sensory Function
**Sticky Dot Test**	Assess Sensorimotor Deficits
**Chemical Stimuli:**
**The Formalin Pain Test**	Biphasic Pain Response
**Mechanical Stimuli:**
**Manual or Electronic Von Frey Test**	Stimulus Intensity that Evokes A Withdrawal Reflex
**The Randall Selitto Test**	Measurement of Pain Response
**Heat Stimuli:**
**Tail Flick Test**	Test for Pain Response
**Hot Plate Test**	Test for Pain Response
**Hargreaves Test**	Assess Thermal Pain Sensation
**Thermal Probe**	Test to Quantify Heat Thresholds
**Cold Stimuli:**
**Cold Plate Test**	Understanding Cold Allodynia and Hyperalgesia
**Acetone Evaporation**	Test to Cold Allodynia
**Cold Plantar Assay**	Assessment of Cold Sensitivity
**Tests For Escape Behavior:**
**Paw Withdrawal Test**	Pain Leading to Escape Reaction
**Acoustic Startle**	Reflex Response to Sudden Loud Sound
**Sensorimotor Gating**	Pre-pulse Inhibition- Weak Stimulus Suppress the Startle Response

**Table 3:** List of various methods in testing cognitive (learning and memory) functions of rodents

**Name of the Behavioural Test**	**To Identify**
**Morris Water Maze Test**	Spatial Learning and Memory
**Radial Arm Maze Test**	
**Modified Elevated Plus-Maze Test**	Spatial Learning
**Three-Panel Runway Maze**	Working Memory
**Y-Maze Test**	Active Working Short-Term Memory
**Barnes Maze**	Working and Spatial Memory Acquisition
**T-Maze Test**	Acquistion, Short-Term Memory, Learning
**8-Arm Radial Maze Test**	Short-Term and Spatial Memory Testing
**Y Maze Forced Alternation Task**	Exploratory Behaviour and Working Memory
**Radial Arm Water Maze Test**	Spatial and Working Reference Memory
**Y-Maze Spontaneous Alternation Task**	Spatial Working Memory, Learning to Explore New Environment
**Star Maze**	Spatial Learning
**Cincinnati Water Maze**	Measuring Escape Latency
**Multiple T-Maze**	Memory and Spatial Learning
**Complex Alley Maze**	Exploratory, Learning Behaviour
**Cheeseboard Maze**	Assess Spatial Learning and Memory
**Hole Board Discrimination Test**	Spatial Working and Reference Memory Performance
**Operant Chamber**	Learned Performance Short-Term Memory
**Passive Avoidance Test**	Avoidance Learning
**Novel Object Recognition Tests**	Associative Memory, Recognition Memory, Declarative Memory, Working Memory
**Swimming Pool Spatial Tasks**	Spatial Behavior
**Fear Conditioning**	Associative Memory
**Hippocampal Working Memory Test**	Spontaneous Alternation in Y-Maze
**Olfactory Learning and Memory Test**	Conditioned Preference for Odor

**Table 4:** List of various methods in assessing emotional state of rodents.

**Tests to Evaluate Emotion, Anxiety and Depression like Behavior: **	
**Open Field**	Anxiety and Exploratory Activity
**Novelty Suppressed Feeding**	Measurement of Depression like Behaviours
**Elevated Plus Maze**	Measurement of Depression like Behaviours
**Light/Dark Box**	Measurement of Anxiety Related Behavior
**Stress Induced Hyperthermia**	Screening for Anxiolytic Potential in Test Compound
**Elevated Zero Maze**	Measurement of Anxiety Related Behavior
**Marble Burying Test**	Measurement of Anxiety Related Behavior
**Hole-Board Test**	Measurement of Anxiety, Stress And Emotionality
**Sucrose Preference Test**	Indicator of Anhedonia
**Tail Suspension Test**	Measurement of Stress
**Forced Swim Test**	Models of Depressive like Behavior
**Coated Test**	Decreased Self-Care - Depression-like Behavior
**Splash Test**	Reduced Grooming Behavior - Depression
**Nesting Test**	Assessing Behavior Associated with Depression
